# A Self-Deliverable H_2_O_2_-Responsive Tocopherol Dimer for Enhanced Antioxidant and Liposomal Delivery

**DOI:** 10.3390/molecules31071071

**Published:** 2026-03-25

**Authors:** Hanui Jo, Ayoung Kim, Changhee Park, Soyoon Baek, Inki Hong, Mingi Kim, Dongwon Lee

**Affiliations:** 1 Department of Bionanotechnology and Bioconvergence Engineering, Jeonbuk National University, Jeonju 54896, Republic of Korea; 2 R&D Complex, Kolmar Korea, 61, 8-gil, Heolleungro, Seocho-gu, Seoul 06800, Republic of Korea; 3 Department of Cosmetics Engineering, Konkuk University, 120 Neungdongro, Gwangjin-gu, Seoul 05029, Republic of Korea; 4 Business Development Division, ICBIO Inc., Naeyuri 1-gil 1, Ipjang-myeon, Seobuk-gu, Cheonan 31027, Republic of Korea; 5 Department of Polymer-Nano Science and Technology, Jeonbuk National University, Jeonju 54896, Republic of Korea

**Keywords:** tocopherol, hydrogen peroxide, antioxidant, liposome, dimer

## Abstract

Oxidative stress induced by excessive hydrogen peroxide (H_2_O_2_) is a critical pathological factor in skin aging, inflammatory disorders, and photodamage. While tocopherol (TCP) is a gold-standard antioxidant in cosmetics, its potential in H_2_O_2_-responsive systems remains underexplored. In this study, we report the design and characterization of ditocopheryl peroxalate (TOT), a novel tocopherol dimer linked via a H_2_O_2_-cleavable peroxalate linkage. TOT remains chemically stable under physiological conditions but undergoes selective chemiluminescence-like degradation upon exposure to H_2_O_2_, simultaneously scavenging H_2_O_2_ and liberating two TCP molecules. Notably, TOT demonstrated superior H_2_O_2_-scavenging efficiency and enhanced antioxidant and anti-inflammatory effects in H_2_O_2_-stimulated cells compared to monomeric TCP, while maintaining excellent biocompatibility. Structural analysis revealed that the rigid, linear configuration of TOT facilitates seamless integration into dipalmitoylphosphatidylcholine (DPPC) bilayers, yielding highly stable H_2_O_2_-responsive liposomes. These findings highlight TOT as a sophisticated multifunctional antioxidant platform for advanced cosmeceutical applications targeting photo-induced oxidative damage.

## 1. Introduction

Cells continuously generate reactive oxygen species (ROS), including hydrogen peroxide (H_2_O_2_), superoxide anions, and nitric oxide, as byproducts of metabolic processes [1]. While ROS play essential roles in cell proliferation, signaling, and immune responses, their excessive accumulation leads to oxidative stress, causing irreversible damages to lipids, proteins, and DNA [2,3]. Notably, H_2_O_2_ is the most prevalent ROS and, despite its moderate reactivity, acts as a precursor to highly toxic hydroxyl radicals [4]. In the skin, oxidative stress is further exacerbated by light-induced photosensitization of cosmetic ingredients [5], which accelerates lipid peroxidation and DNA fragmentation via Type-I and Type-II mechanisms [6]. Given the detrimental impact of excess ROS, particularly H_2_O_2_, there is an urgent demand for advanced antioxidant strategies that can selectively mitigate H_2_O_2_-induced phototoxicity and skin aging [7].

Tocopherol (TCP) is a family of natural phenols derived from shikimic acid, characterized by a polar chromanol head and isoprenic side chain [8]. There are four different tocopherols (α, β, γ and δ), differing in the number and position of methyl groups on the chromanol head [9]. As a fat-soluble antioxidant essential for human health, TCP protects cell membranes from damage by ROS and plays essential roles in immune function, skin health, and vision [10,11]. Its antioxidant activity is ascribed to the easy hydrogen transfer to the peroxy radical, leading to the production of a tocopheroxy radical, which combines with another lipid peroxy radical to subsequently generate various nonradical tocopherol oxidation products [9,12,13]. TCP is a gold standard in both pharmaceutical and cosmetic applications due to its ability to neutralize lipid peroxidation and enhance skin barrier function [14]. However, the potential of tocopherol-based systems that leverage H_2_O_2_-scavenging mechanisms has yet to be fully realized.

H_2_O_2_-activated prodrugs have been extensively studied in pharmaceutical and biomedical fields for their targeted therapeutic benefits [3,15,16]. Among various stimuli-responsive motifs, the peroxalate linkage has emerged as a premier H_2_O_2_-cleavable moiety, facilitating the controlled activation of prodrugs through oxidative degradation [17,18]. While its utility is well-established in clinical drug delivery, its integration into cosmeceutical formulations remains largely unexplored. To address this gap, we have developed ditocopheryl peroxalate (TOT), a novel H_2_O_2_-cleavable antioxidant, in which two TCP units are bridged via a peroxalate bond. The rationale for employing the peroxalate linkage lies in its exceptional H_2_O_2_ sensitivity and the ability to finely tune its kinetic profile; for instance, the incorporation of electron-withdrawing substituents can accelerate H_2_O_2_-responsiveness, albeit at the potential cost of hydrolytic stability [19]. Exploiting these tunable properties and its excellent biocompatibility, we utilized the peroxalate moiety as the functional core of TOT.

Distinct from conventional antioxidants that act as passive payloads within delivery vehicles, TOT is designed to align within lipid bilayers, allowing for its seamless incorporation as a structural component in liposomal formulations. This unique configuration ensures that the antioxidant itself constitutes the delivery matrix, enhancing loading efficiency and structural integrity. TOT remains robust under physiological conditions but undergoes rapid degradation upon encountering pathological levels of H_2_O_2_, simultaneously scavenging excess H_2_O_2_ and liberating two functional TCP molecules. Furthermore, we demonstrate that TOT effectively protects cutaneous cells from H_2_O_2_-induced oxidative stress and phototoxicity. Herein, we evaluate the physicochemical properties of TOT and highlight its potential as a next-generation active cosmeceutical ingredient for advanced skin protection.

## 2. Results and Discussion

### 2.1. Synthesis and Characterization of TOT

**Figure 1:**
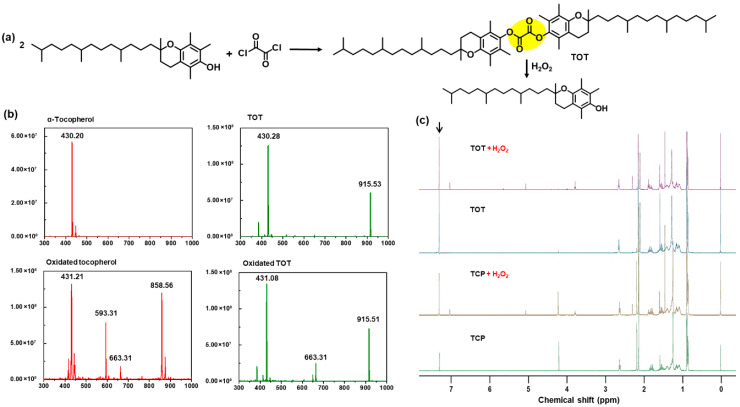
Synthesis and characterization of TOT. (**a**) A synthetic route and degradation of TOT. (**b**) LC/MS MS chromatography of TCP, TOT and oxidation product of compounds. (**c**) ^1^H-NMR spectroscopy of TCP, TOT and TCP and TOT oxidation products in CDCl_3_.

TOT was designed as a H_2_O_2_-responsive dimer of TCP by exploiting the high reactivity of peroxalate linkages toward nucleophilic oxygen species [20,21,22]. TOT synthesis was achieved through a straightforward esterification between the hydroxyl group of TCP and oxalyl chloride (Figure 1a). Following purification, TOT was obtained as a waxy solid, a distinct physical transformation from the naturally oily state of monomeric TCP. The chemical structure of TOT was rigorously validated using NMR and mass spectroscopy. Specifically, the disappearance of the phenolic hydroxy proton signal (~4.2 ppm) and the characteristic downfield shift of the adjacent methyl protons (~2.2 ppm) confirmed the successful formation of the peroxalate-bridged dimer TOT (Figure S1).

We next examined the H_2_O_2_-triggered degradation profile of TOT to verify its potential as an antioxidant. LC-MS/MS chromatography revealed that H_2_O_2_ treatment led to TOT degradation, liberating TCP (Figure 1b). In an aqueous environment, TCP underwent further H_2_O_2_-mediated oxidation, forming various metabolites such as tocopheryl quinone [9,11]. This sequential process was evidenced by the evolution of chemical shifts at 2.2 ppm corresponding to methyl protons on the chromanol ring and the significant decrease in the proton signal corresponding to the hydroxy proton (4.2 ppm) (Figure 1c).

The formation of oxidized TCP was also confirmed by MS spectroscopy. TOT treated with H_2_O_2_ exhibited the same proton signal as H_2_O_2_-treated TCP. These findings suggest that TOT degrades in a H_2_O_2_-triggered manner to release TCP. For comparative studies, we also synthesized TOH as a control, integrating TCP and hexadecanol via a peroxalate linker to assess the impact of the dimeric configuration (Figure S2).

### 2.2. H_2_O_2_-Responsiveness of TOT

**Figure 2:**
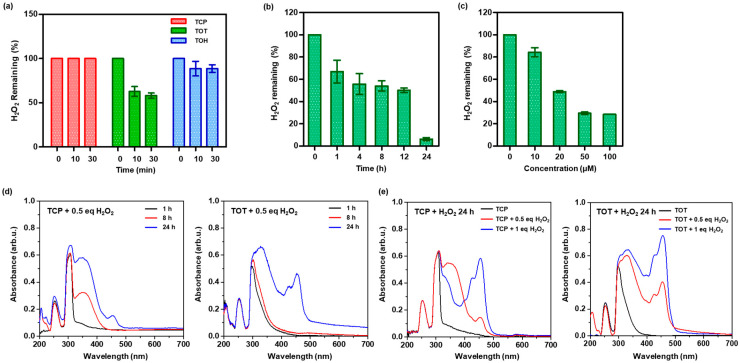
H_2_O_2_-responsiveness of TOT. (**a**) Time-dependent initial H_2_O_2_-scavenging kinetics evaluated in the presence of excess H_2_O_2_ (3 equivalents). (**b**) Time-dependent H_2_O_2_-scavenging profile of TOT (100 μM). (**c**) Concentration-dependent H_2_O_2_-scavenging profile of TOT over 24 h. Values are mean ± s.d. (*n* = 3). (**d**) UV-vis spectroscopy spectrum of TCP and TOT oxidation products according to time (incubated at 30 °C with stirring). (**e**) UV-vis spectroscopy spectrum of TCP and TOT oxidation products by different concentrations of H_2_O_2_ (incubated at 30 °C with stirring).

H_2_O_2_ itself is not highly reactive with biological molecules, but it is a precursor of the hydroxyl radical that is considered the most toxic to cells [4,23]. We therefore assessed the H_2_O_2_-scavenging ability of TOT as a primary indicator of antioxidant performance. To simulate severe oxidative stress and verify whether TOT can rapidly intercept this precursor before downstream radicals form, the initial scavenging kinetics were evaluated using a 3-fold molar excess of H_2_O_2_. Upon the addition of TOT to the H_2_O_2_ solution, its H_2_O_2_ scavenging kinetics were compared directly with those of monomeric TCP and the control dimer TOH (Figure 2a). While TCP exhibited a negligible impact on the level of H_2_O_2_, TOT demonstrated potent, time-dependent H_2_O_2_ scavenging activity even against this excessive oxidative stress, achieving a 40% reduction within 30 min. To further elucidate the structure-activity relationship of the peroxalate linkage, TOH, which integrates TCP and hexadecanol via peroxalate, was used as a control. TOT showed a significantly superior H_2_O_2_-scavenging efficiency compared to TOH. This disparity is attributed to the distinct electronic environments surrounding the peroxalate bridge; TOT possesses two extensive chroman rings (aryl groups), whereas TOH is asymmetric with both aryl and alkyl substituents. Previous studies have established that electron-withdrawing moieties adjacent to peroxalate linkages accelerate H_2_O_2_-mediated cleavage [21,22]. Compared to the aliphatic alkyl chain in TOH, the extensive aromatic rings in TOT act as relative electron sinks. This structural feature increases the electrophilicity of the peroxalate carbonyls, facilitating nucleophilic attack by H_2_O_2_ and thereby enhancing the scavenging efficiency of TOT. Furthermore, TOT displayed both time- and concentration-dependent H_2_O_2_ scavenging profiles (Figure 2b,c).

The H_2_O_2_-responsiveness of TOT was further corroborated by UV-vis spectroscopy. To monitor and compare their distinct oxidation behaviors, both TCP and TOT were reacted with H_2_O_2_ under identical mild conditions (30 °C, constant stirring). As an oxygen donor, H_2_O_2_ oxidizes TCP into a variety of products, including tocopheryl quinone, tocopheryl hydroquinone, and dimers [9]. The H_2_O_2_-induced oxidation was characterized by an increase in UV absorption at ~350 nm and ~450 nm [13]. The absorption band at ~350 nm corresponds to the formation of tocopherol quinone and extended quinone systems, while the distinct and strong absorption band at ~450 nm suggests the generation of highly conjugated quinoid dimers, consistent with our MS analysis (Figure 1b). We first examined the time course of oxidation induced by 0.5 eq. H_2_O_2_ (Figure 2d). Under these mild conditions, TCP exhibited slow and limited spectral changes initially, reflecting its sluggish direct reactivity toward H_2_O_2_. In sharp contrast, TOT underwent much faster H_2_O_2_-mediated oxidation, demonstrating distinct and progressive spectral alterations. Furthermore, both TCP and TOT showed H_2_O_2_-concentration-dependent oxidation (Figure 2e). Compared to TCP, TOT consistently exhibited stronger absorption bands at ~350 nm and ~450 nm, which was also visually supported by a more intense yellowish color (Figure S4). The significantly stronger and faster UV response of TOT can be attributed to its structural design. Given that TOT contains two TCP units, its cleavage generates a higher density of quinone-like intermediates. More importantly, this kinetic superiority is driven by the highly reactive peroxalate linkage, which rapidly cleaves upon reacting with H_2_O_2_ and accelerates the overall oxidation process compared to the kinetically slow direct oxidation of monomeric TCP. These findings conclusively suggest that TOT functions as a highly effective, H_2_O_2_-triggered antioxidant platform with superior kinetic advantages over conventional TCP.

### 2.3. Cytoprotective and Anti-Inflammatory Effects of TOT

**Figure 3:**
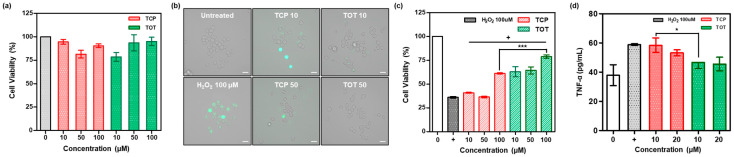
Biological activities of TOT. (**a**) Cytotoxicity of against RAW 264.7 cells. (**b**) Fluorescence images of H_2_O_2_-stimulated RAW264.7 cells. The cells were stained with DCFH-DA as a ROS probe (green fluorescence). Scale bar: 20 μm. (**c**) Cytoprotective effects of TOT on H_2_O_2_-simulated RAW 264.7 cells. (**d**) The level of TNF-α in H_2_O_2_-stimulated RAW 264.7 cells. The ‘+’ sign indicates uniform treatment with H_2_O_2_ across the respective group. Values are mean ± s.d. (*n* = 3). * *p* < 0.05, *** *p* < 0.001.

To evaluate the potential of TOT as a high-performance cosmeceutical ingredient, its biocompatibility was first assessed using an MTT assay in RAW264.7 macrophages [24]. The experimental concentrations of TCP and TOT were carefully determined based on preliminary toxicity tests, the optimal reactive range observed in our earlier chemical H_2_O_2_-scavenging assays, and relevant previous literature on tocopherol-based antioxidants. Figure 3a shows the viability of RAW264.7 cells treated with TCP and TOT for 24 h. TOT had a negligible impact on cell viability at concentrations up to 100 μM, suggesting its excellent biocompatibility and establishing 100 μM as the safe upper limit for subsequent in vitro efficacy tests.

The intracellular antioxidant capacity of TOT was then scrutinized in H_2_O_2_-stimulated RAW264.7 cells using DCFH-DA, which is a cell-permeable fluorogenic probe commonly used to detect ROS [25]. Exposure to H_2_O_2_ triggered a robust generation of intracellular ROS, manifested by intense green fluorescence throughout the cytoplasm (Figure 3b). While monomeric TCP reduced H_2_O_2_-mediated ROS levels in a concentration-dependent manner, TOT demonstrated a significantly more potent suppression of ROS generation within the selected non-toxic concentration range. This enhanced efficacy is likely attributed to the synergistic interplay between the H_2_O_2_-scavenging peroxalate linkage and the liberated TCP molecules, which provide a dual-stage defense against oxidative stress.

We also examined the cytoprotective effect of TOT against H_2_O_2_-mediated toxicity. Incubation with 100 μM H_2_O_2_ significantly impaired cell viability; however, pretreatment with TOT effectively rescued cells from this cytotoxicity (Figure 3c). TOT effectively protected cells from H_2_O_2_-mediated cytotoxicity at significantly lower concentrations. Notably, 10 μM of TOT exhibited cytoprotective effects comparable to 100 μM of TCP, indicating a ten-fold increase in protective efficiency. We next examined the anti-inflammatory effects of TOT by measuring the level of TNF-α, which is one of the key pro-inflammatory cytokines involved in chronic inflammation. H_2_O_2_-stimulated cells showed a markedly elevated level of TNF-α (Figure 3d). While monomeric TCP exhibited little to no effect on the level of TNF-α, TOT treatment resulted in a significant, dose-dependent inhibition of TNF-α expression. These observations collectively underscore that TOT exerts potent, multi-targeted antioxidant and anti-inflammatory activities, effectively mitigating the pathological signals induced by excessive H_2_O_2_.

### 2.4. Liposomal Formulation of TOT

**Figure 4:**
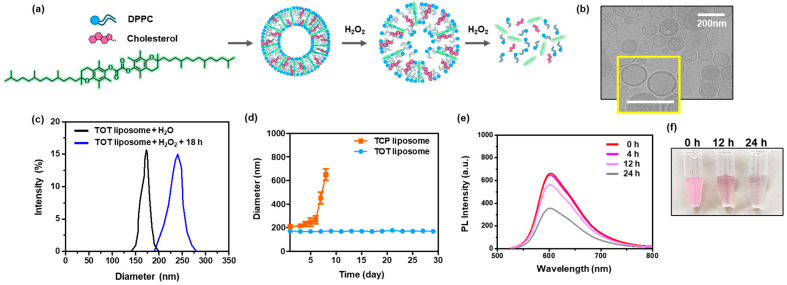
Characterization of TOT-containing liposomes. (**a**) Schematic illustration of TOT-containing liposomes with H_2_O_2_-responsiveness. (**b**) Representative Cryo-TEM image of TOT-containing liposomes. (**c**) Representative dynamic light scattering of TOT-containing liposomes treated with H_2_O_2_ for 18 h. (**d**) Changes in diameter of TOT-containing liposomes over 30 days of observation period. (**e**) Changes in fluorescence emission spectra of Nile Red-loaded TOT-containing liposomes in the presence of H_2_O_2_. (**f**) Photographs of Nile Red-loaded TOT-containing liposomes. The yellow box indicates a magnified view of the highlighted region.

Despite its potent antioxidant activity, the clinical and cosmeceutical applications of TCP have been limited by its low water solubility and premature oxidation [12]. Phospholipid liposomes have been widely used to improve its solubility and enhance delivery efficacy into cells and tissues. TCP is known to bind strongly with lipids, possibly through hydrogen bond formation between the hydroxyl group of the former and one of the oxygen atoms of the latter [26]. However, the low solubility of TCP in the aqueous core leads to an uneven distribution within the liposomal layer and makes encapsulation inefficient. Additionally, oily TCP disrupts the liposomal membrane, leading to leakage or rupture of the liposomes during long-term storage. In this regard, the composition and TCP content should be carefully adjusted to enhance the integrity of liposomes and reduce leakage. Based on the rationale that waxy TOT has a higher transition temperature than TCP (Figure S5) and therefore facilitates a more stable integration into the DPPC matrix, we engineered TOT-integrated liposomes (DPPC/cholesterol/TOT = 6:2:2 weight ratio) (Figure 4a). The resulting TOT-containing liposomes formed uniform spherical vesicles with a mean hydrodynamic diameter of ~180 nm, as confirmed by dynamic light scattering and electron microscopy (Figure 4b,c). We also tested the stability of liposomes containing TCP and TOT. The colloidal stability of TOT-containing liposomes was assessed by monitoring their hydrodynamic diameter over 30 days of incubation in the phosphate buffer (Figure 4d). The TOT-containing liposomes maintained their initial size for over 30 days in the phosphate buffer, whereas TCP-containing liposomes displayed a sharp increase in diameter after just 6 days, suggesting that the rigid, linear structure of TOT promotes seamless alignment with the hydrophobic tails of DPPC through enhanced van der Waals interactions, thereby reinforcing the bilayer integrity.

We also examined the H_2_O_2_-responsiveness of TOT-containing liposomes. After 18 h of H_2_O_2_ treatment, the hydrodynamic diameter of TOT-containing liposomes markedly increased, indicating that the H_2_O_2_-mediated cleavage of the peroxalate ester in TOT triggers bilayer disruption and subsequent liposomal reorganization. To further substantiate the H_2_O_2_-responsiveness of TOT-containing liposomes, we used Nile Red as a fluorescent probe, which exhibits strong red fluorescence in lipid-rich environments, while typically remaining non-fluorescent in hydrophilic environments. Nile Red-loaded liposomes exhibited a robust emission peak at 590 nm (Figure 4e), confirming its sequestration within the hydrophobic bilayer. However, in the presence of H_2_O_2_, the fluorescence intensity gradually decreased with time (Figure 4f), indicating that Nile Red is released from the liposome and loses fluorescence. We also tested the stability and H_2_O_2_-responsiveness of TOH-liposomes for comparison purposes. TOH-containing liposomes exhibited a drastic change in hydrodynamic diameter from day 7 (Figure S6a). The hydrodynamic diameter rapidly increased upon the addition of H_2_O_2_ and continued to gradually increase over the 1 h of observation period (Figure S6b). The rapid H_2_O_2_-responsiveness of TOH-containing liposomes could be attributed to their inferior colloidal stability, which induces the rapid permeation of H_2_O_2_. These findings highlight that the dimeric, aryl-rich structure of TOT is essential not only for kinetic reactivity but also for maintaining the structural architecture of responsive cosmeceutical delivery systems.

### 2.5. Cellular Internalization and Biological Activity of TOT-Containing Liposomes

**Figure 5:**
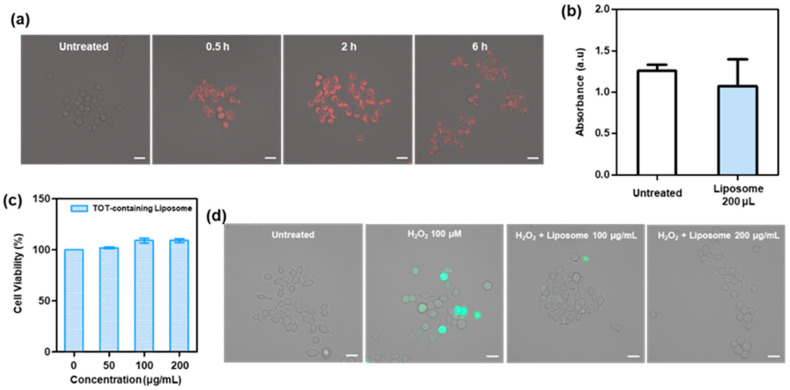
Biological activity of TOT-containing liposomes. (**a**) Fluorescence images of RAW264.7 cells treated with Nile Red-loaded TOT liposomes (red fluorescence). Scale bar: 20 μm. (**b**) The level of LDH released from cells treated with TOT liposomes (200 μg/mL). Values are mean ± s.d. (*n* = 3). (**c**) Viability of RAW 264.7 cells treated with TOT liposomes. Values are mean ± s.d. (*n* = 3). (**d**) Fluorescence images showing the intracellular level of ROS in H_2_O_2_-stimulated RAW264.7 cells. Cells were stained with DCFH-DA (green fluorescence). Scale bar: 20 μm.

TCP acts as a potent antioxidant primarily by scavenging ROS that can damage cells. This process occurs within the cellular environment, particularly in cell membranes, where TCP prevents oxidative damage to lipids and proteins. For bioactive substances like TOT to exert their antioxidant effects and modulate inflammatory pathways efficiently, they generally need to be internalized into cells. We therefore investigated the cellular uptake kinetics of TOT-containing liposomes in RAW264.7 cells using Nile Red as a membrane-mimetic fluorescent probe [27]. As shown in Figure 5a, distinct red fluorescence was observed in the cell membranes and throughout the cytosol as early as 30 min post-incubation. The fluorescence intensity increased over time, suggesting that the liposomes are successfully internalized via endocytic pathways while maintaining their structural integrity. Notably, at 6 h post-incubation, a decline in fluorescence intensity was observed, indicating the programmed disruption of liposomes and the subsequent release of Nile Red in the relatively hydrophilic environment of the cytosol.

Given that liposomal formulations can induce unintended cytotoxicity through plasma membrane disruption, we performed a lactate dehydrogenase (LDH) leakage assay to evaluate membrane integrity. RAW264.7 cells incubated with TOT-containing liposomes for 12 h showed no significant LDH release, indicating that the formulation does not cause discernible membrane damage (Figure 5b). Additionally, TOT-containing liposomes exhibited negligible effects on the cell viability up to 200 μg/mL after 24 h of incubation (Figure 5c), underscoring their excellent biocompatibility for cosmeceutical use. As expected from Figure 2b, TOT-containing liposomes suppressed the ROS generation in H_2_O_2_-stimulated cells (Figure 5d). These results demonstrate that TOT-containing liposomes not only facilitate the efficient delivery of antioxidants into the cellular interior but also provide a robust defense against oxidative stress through their unique H_2_O_2_-responsive degradation mechanism.

## 3. Materials and Methods

### 3.1. Synthesis and Structural Analysis of TOT

TOT was synthesized via the reaction of TCP and oxalyl chloride. Briefly, oxalyl chloride (4.64 mmol) was dissolved in dichloromethane (DCM) and stirred in an ice-water bath for 30 min. Then, TCP (9.28 mmol) dissolved in DCM was added dropwise while maintaining the temperature at 0 °C. After a 2 h reaction, the mixture was purified by silica gel chromatography using hexane/ethyl acetate (7:1, *v*/*v*) as the eluent. TOT was obtained as a yellow wax (70% yield), and its chemical structure was confirmed by 500 MHz ^1^H and ^13^C NMR spectroscopy (JNM-EX500, JEOL, Akishima, Japan) using CDCl_3_ as the solvent [19]. The transition temperature of TOT was determined using a differential scanning calorimeter (TA Instruments, New Castle, DE, USA, Discovery DSC2500).

### 3.2. Synthesis of α-Tocopherol-Derivative-Containing Hexadecanol (TOH)

TOH was synthesized in two steps. In the initial step of the synthesis, hexadecanol was dissolved in anhydrous diethyl ether and subsequently reacted with an excess amount of oxalyl chloride under ice-bath conditions (0 °C), leading to the formation of a monoester-acyl chloride intermediate. The crude reaction mixture was then subjected to liquid–liquid extraction using water and diethyl ether. During this work-up, the remaining acyl chloride moiety was selectively hydrolyzed to the corresponding carboxylic acid, yielding the monoester-monoacid product. In the subsequent step, the monoester-monoacid obtained from the previous reaction was dissolved in DCM, followed by the addition of α-tocopherol (1.5 eq.), 1-ethyl-3-(3-dimethylaminopropyl)carbodiimide (EDC, 4.0 eq.), and dimethylaminopyridine (DMAP, 0.4 eq.). The reaction mixture was stirred at room temperature overnight to allow esterification. Upon completion, the mixture was washed with distilled water (2–3 times) to remove water-soluble byproducts. The organic phase was dried over anhydrous sodium sulfate (Na2SO_4_), filtered, and concentrated under reduced pressure. The crude product was then purified by column chromatography using hexane/ethyl acetate (7:1, *v*/*v*) as the eluent, yielding the final ester compound (20% yield). Chemical structure was identified using 500 MHz ^1^H and ^13^C NMR spectroscopy (JNM-EX500 JEOL, Akishima, Japan) using CDCl_3_ as a solvent.

### 3.3. H_2_O_2_-Induced Oxidation of TCP and TOT

To investigate H_2_O_2_-induced oxidative modifications of TCP and TOT, each compound was dissolved in tetrahydrofuran (THF) at a concentration of approximately 3 mM under stirring at 30 °C. Equimolar amounts of H_2_O_2_ were added, and the solvent was evaporated under reduced pressure. The residues were dissolved in deuterated CDCl_3_ and analyzed by ^1^H NMR spectroscopy to confirm the structural changes. Additionally, liquid chromatography-tandem mass spectrometry (LC/MS/MS) was performed to identify oxidation products and infer degradation pathways based on fragmentation patterns [9,11].

### 3.4. UV-Vis Spectroscopy of TOT

To evaluate the structural changes upon H_2_O_2_ exposure, both TCP and TOT solutions were incubated with H_2_O_2_ under continuous stirring at 30 °C. The UV-vis absorption spectra were then recorded at predetermined time intervals. The UV-Vis absorption spectra were recorded using a spectrophotometer (S-3100, Scinco, Seoul, Republic of Korea) with a 1 cm quartz cuvette. TOT was dissolved in THF, and spectra were acquired at room temperature over the wavelength range of 200~800 nm in the presence or absence of H_2_O_2_. THF served as both the solvent and blank.

### 3.5. H_2_O_2_ Scavenging Assay

The H_2_O_2_ scavenging ability of TOT was evaluated using a peroxalate-based chemiluminescence assay [17,18]. A fluorescent working solution containing rubrene, diphenyloxalate (DPO), and THF was used as the detection system. Upon reaction with H_2_O_2_, DPO produces a high-energy intermediate that excites rubrene, emitting detectable light. TOT or TCP (used as a reference antioxidant) was dissolved in THF and added at varying concentrations (5, 10, 20, 50, and 100 μM) to a fixed 100 μM H_2_O_2_ solution. Residual H_2_O_2_ levels were quantified by measuring chemiluminescence intensity using a luminometer. To evaluate the rapid initial kinetics, TOT was incubated with an excess of H_2_O_2_ (3 equivalents) and monitored for 10 min. For time-dependent evaluation, 100 μM of TOT was incubated with 100 μM H_2_O_2_, and luminescence was measured at 0, 1, 4, 8, 12, and 24 h.

### 3.6. Cell Viability Assay

RAW264.7 murine macrophage cells were purchased from the Korea Cell Line Bank (Seoul, Korea) and cultured in a humidified incubator with 5% CO_2_ at 37 °C. Cells were seeded into 24-well plates at a density of 2 × 10^5^ cells/well and cultured to ~80% confluency. Cells were treated with TCP or TOT at 10, 20, 50, and 100 μM for 24 h. After the treatment, 100 μL of MTT solution was added to each well, followed by a 3 h incubation [24]. One milliliter of dimethyl sulfoxide (DMSO) was added to each well to dissolve the formazan crystals, and absorbance was measured at 570 nm using a microplate reader (Synergy MX, BioTek, Winooski, VT, USA). For oxidative stress protection, cells were pretreated with 100 μM H_2_O_2_ for 30 min, followed by antioxidant treatment.

### 3.7. TNF-α Secretion by ELISA

RAW264.7 cells were seeded at ~2 × 10^5^ cells/well and incubated with TCP or TOT (10 or 20 μM). After 24 h of incubation, culture media were collected, centrifuged at 10,000× *g* for 10 min, and analyzed using a Mouse TNF-α ELISA kit (Thermo Fisher Scientific, Waltham, MA, USA) according to the manufacturer’s instructions. Absorbance was read at 450 nm and cytokine levels were calculated from a standard curve.

### 3.8. Formulation and Characterization of TOT-Containing Liposomes

Liposomes were prepared using DPPC, cholesterol, and TOT at a 6:2:2 (*w*/*w*/*w*) ratio (a total of 10 mg) [26]. DPPC and cholesterol were dissolved in ethanol, and TOT in ethyl acetate. The solutions were combined to a 1 mL total volume and incubated at 40 °C in a bath sonicator for 30 min. One milliliter of distilled water was added gradually with stirring. The mixture underwent rotary evaporation under reduced pressure to remove residual organic solvents, yielding liposomes at a concentration of 10 mg/mL. A mini-extruder system was used to obtain uniformly sized liposomes. Cryo-TEM was performed to observe the morphology of the liposome under near-native conditions. Samples were first vitrified to prevent ice crystal formation by rapidly freezing them via plunge freezing in liquid nitrogen. The vitrified samples were then applied onto specialized cryo-TEM grids. Cryo-TEM analysis was performed at the Center for University Joint Research Facilities, Jeonbuk National University. The hydrodynamic size and polydispersity index (PDI) of TOT-containing liposomes were measured by dynamic light scattering using a particle size analyzer (Brookhaven Instrument Corp., Holtsville, NY, USA). For fluorescence imaging and photoluminescence Nile Red (1.0 wt%) was loaded in TOT-containing liposomes. Nile Red was added in ethanol containing DPPC and cholesterol. The following procedure was the same as that for the TOT-containing liposomes. The fluorescence emission of Nile Red-liposomes was obtained using a spectrofluorometer (FP6500 Jasco, Hachioji, Japan) in the presence and absence of H_2_O_2_.

### 3.9. Fluorescence Imaging

To evaluate the ability of TOT and TOT-containing liposomes to suppress oxidative stress, cells were seeded in a confocal dish (SPL life Sciences, Seongnam, Korea) at a 6 × 10^4^ cells per dish. Liposomes and TOT at various concentrations were added in each dish and incubated for 24 h. After gentle washing with PBS, the cells were treated with 20 μM of 2′,7′-dichlorofluorescein-diacetate (DCFH-DA) dissolved in DMSO for 30 min to determine the level of intracellular ROS [25]. Cells were washed with PBS and observed under the confocal laser scanning microscope (CLSM, Carl Zeiss, Jena, Germany) to determine the level of intracellular ROS. To observe the internalization of TOT-containing liposomes, cells seeded in a confocal dish at 6 × 10^4^ cells were treated with Nile red-loaded liposomes for 0.5, 2 or 6 h and washed with PBS.

### 3.10. LDH Cytotoxicity Assay

The cells seeded in 6-well plates were incubated with TOT-containing liposomes for 24 h. After treatment, cells were lysed and centrifuged at 600× *g* for 5 min. Supernatants were analyzed using a WST-based LDH detection kit (Dojindo, Kumamoto, Japan). A reaction mixture was prepared by mixing the WST Substrate Mix with the Assay Buffer, and 100 μL was added to each well in a 96-well plate. Plates were incubated for 30 min at room temperature in the dark. Absorbance was measured at 450 nm using a microplate reader (Synergy MX, BioTek, Winooski, VT, USA).

## 4. Conclusions

We have successfully developed ditocopheryl peroxalate (TOT), a novel H_2_O_2_-responsive antioxidant dimer designed to overcome the structural and functional limitations of conventional tocopherol (TCP). By integrating a chemiluminescent-inspired peroxalate linkage, TOT functions as a high-performance molecular switch that selectively undergoes degradation in the presence of H_2_O_2_. This programmed response enables a dual-action defense mechanism: the simultaneous scavenging of toxic excessive H_2_O_2_ and the site-specific liberation of two active TCP molecules. Beyond its chemical reactivity, the rigid and linear configuration of TOT facilitates superior integration into phospholipid bilayers, yielding a H_2_O_2_-responsive liposomal formulation with enhanced colloidal stability compared to monomeric TCP. Cell culture studies revealed that TOT-containing liposomes were efficiently internalized via endocytosis and exhibited excellent biocompatibility, providing more potent antioxidant and anti-inflammatory protection in H_2_O_2_-stimulated cells. Collectively, our results demonstrate that TOT offers superior protective effects over native TCP and holds great potential as an active cosmeceutical ingredient for oxidative stress mitigation. Although this study establishes the robust H_2_O_2_-scavenging kinetics of TOT in solution, its high hydrophobicity limits direct application in aqueous biological media. Therefore, future research will focus on developing water-dispersible formulations of TOT, such as nanocarriers, to evaluate its antioxidant efficacy in complex in vitro cellular models.

## Data Availability

Data is contained within the article and the Supplementary Materials.

## Supplementary Materials

The following supporting information can be downloaded at: https://www.mdpi.com/article/10.3390/molecules31071071/s1, Figure S1: Characterization of TOT; Figure S2: Synthetic route of TOH; Figure S3: Characterization of TOH; Figure S4: Photographs of TCT and TOT after H_2_O_2_ treatment; Figure S5: DSC thermogram of TOT; Figure S6: Change in hydrodynamic diameter of TOH-containing liposomes.
