# Supplementary material for: A Self-Deliverable H2O2-Responsive Tocopherol Dimer for Enhanced Antioxidant and Liposomal Delivery
Source: Molecules. 2026 Mar 25;31(7):1071. doi: 10.3390/molecules31071071 (PMC13074395; doi:10.3390/molecules31071071)
Supplement: Supplementary file 1 [file molecules-31-01071-s001.zip › molecules-4189021-supplementary.pdf]

Supporting Information

A Self-Deliverable H_2O_2-Responsive Tocopherol Dimer for Enhanced Antioxidant and Liposomal Delivery

Hanui Jo [1], Ayoung Kim [2], Changhee Park [2], Soyoon Baek [2,3], Inki Hong [2], Kim Mingi [4] and Dongwon Lee [1,5,*]

[1] Department of Bionanotechnology and Bioconvergence Engineering, Jeonbuk National University, Jeonju, Jeonbuk 54896, Republic of Korea
[2] R&D Complex, Kolmar Korea, 61, 8-gil, Heolleungro, Seocho-gu, Seoul 06800, Republic of Korea
[3] Department of Cosmetics Engineering, Konkuk University, 120 Neungdongro, Gwangjin-gu, Seoul 05029, Republic of Korea
[4] Business Development Division, ICBIO, Naeyuri 1-gil 1, Ipjang-myeon, Seobuk-gu, Cheonan, Chungnam 31027, Republic of Korea
[5] Department of Polymer–Nano Science and Technology, Jeonbuk National University, Jeonju, Jeonbuk 54896, Republic of Korea
* Correspondence: dlee@jbnu.ac.kr; Tel.: +82-63-270-2344

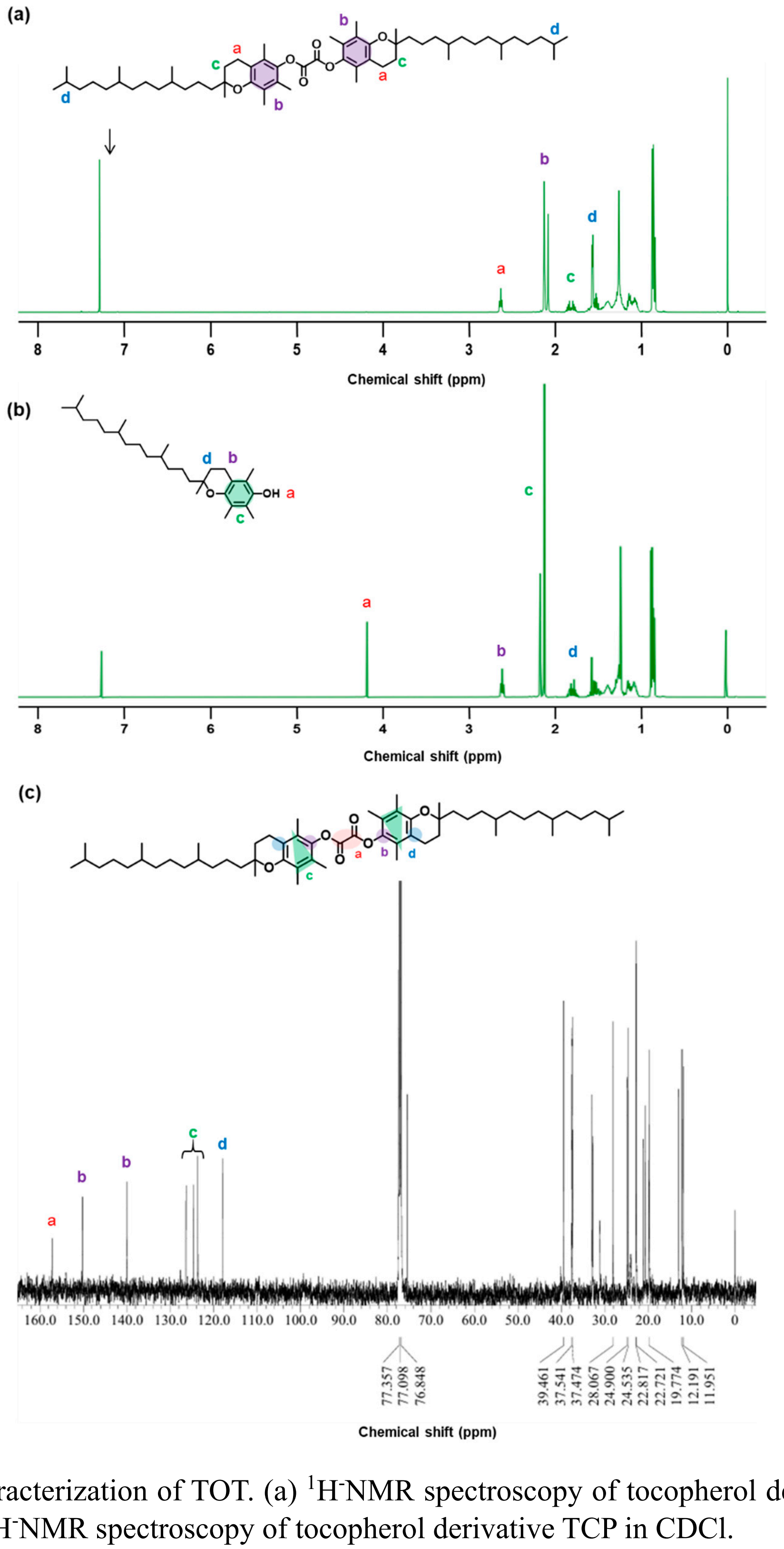

Figure S1. Characterization of TOT. (a) ^{1}H-NMR spectroscopy of tocopherol derivative TOT in $CDCl_3$. (b) ^{1}H-NMR spectroscopy of tocopherol derivative TCP in CDCl.

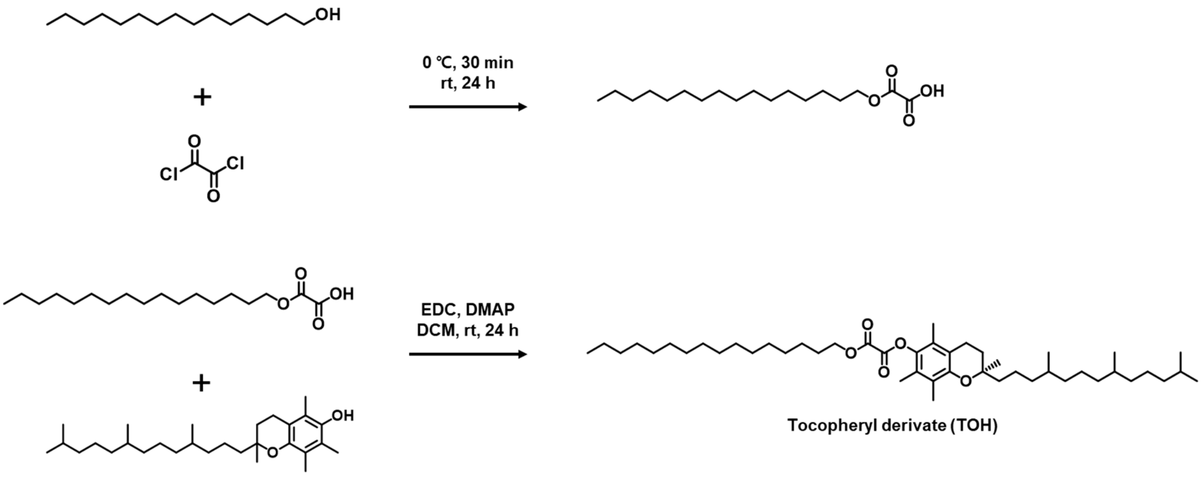

Figure S2. Synthetic route of TOH.

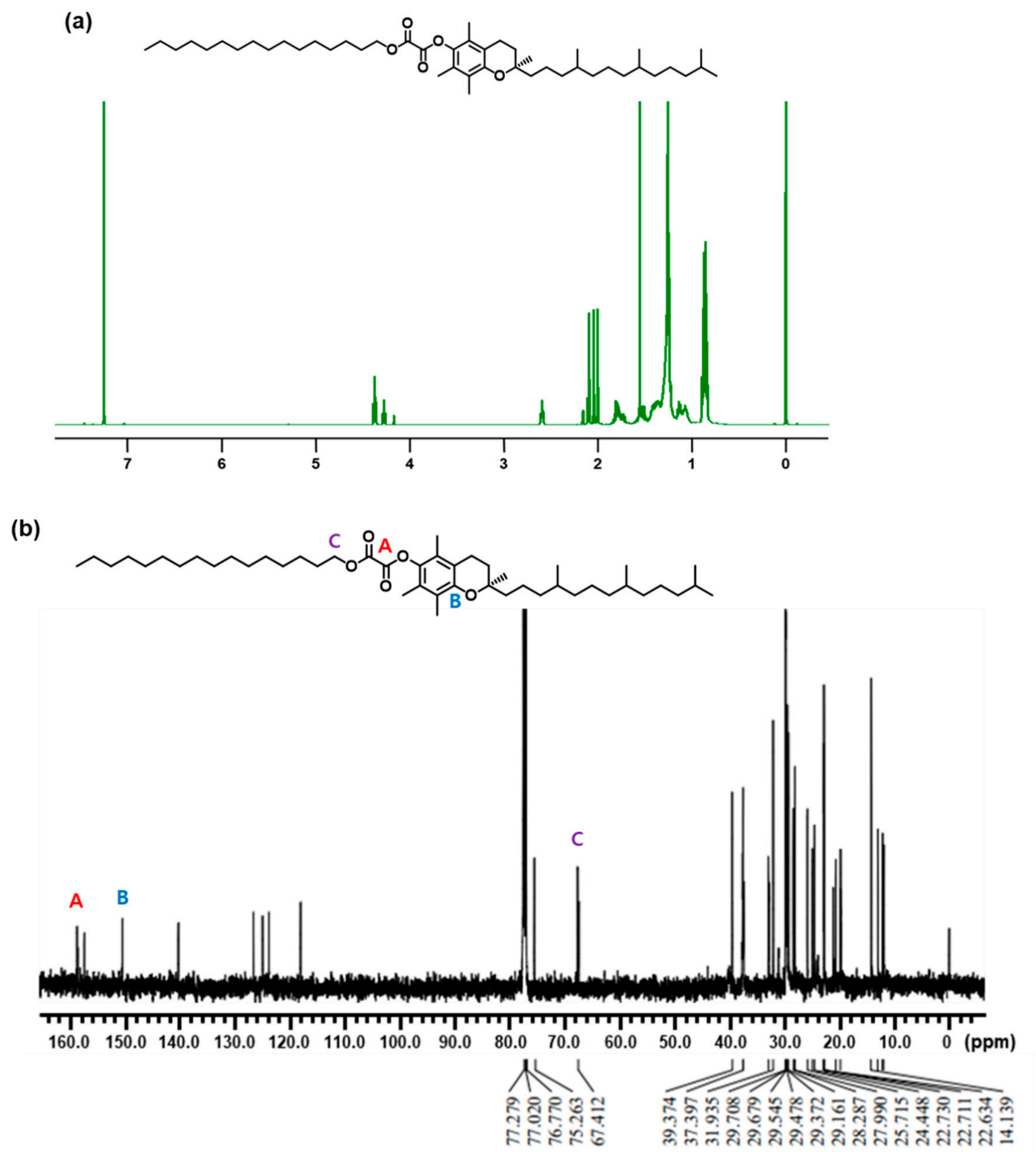

Figure S3. Characterization of TOH. (a) ^{1}H-NMR spectroscopy of tocopherol derivative TOT in $CDCl_3$. (b) ^{1}H-NMR spectroscopy of tocopherol derivative TCP in $CDCl_3$.

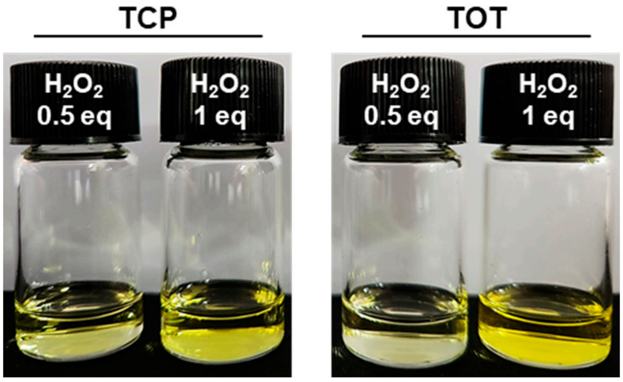

Figure S4. Photographs of TCT and TOT after H_2O_2 treatment.

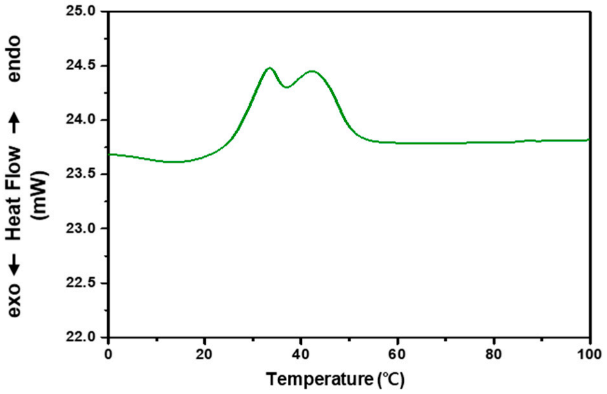

Figure S5. DSC thermogram of TOT.

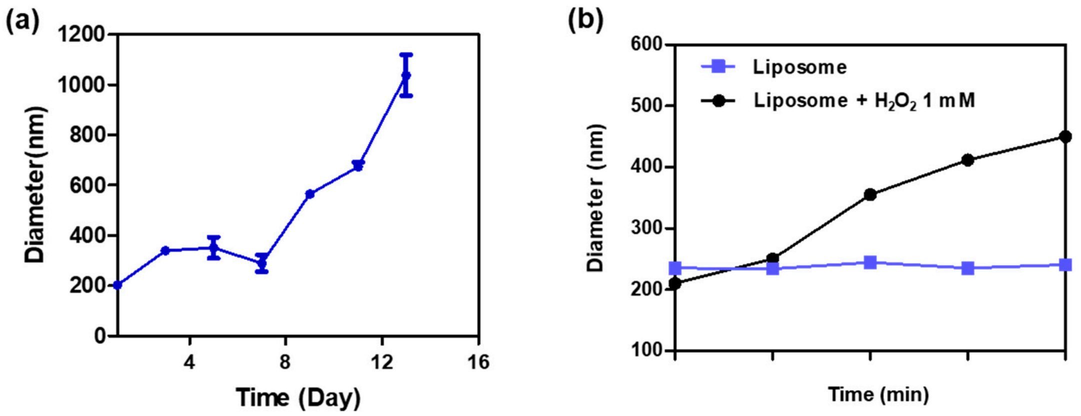

Figure S6. Change in hydrodynamic diameter of TOH-containing liposomes. (a) Changes in diameter with time. (b) Change in diameter of liposomes in the presence and absence of H_2O_2.
